# High Prevalence of Bone Marrow Involvement and Advanced Disease in Saudi Patients Diagnosed With Hodgkin Lymphoma

**DOI:** 10.7759/cureus.19494

**Published:** 2021-11-12

**Authors:** Musa F Alzahrani, Mohammed B Alkahil, Abdulaziz A Alhusainy, Abdulmohsen K Alangari, Mohammed N Almania, Essam H Alshahrani, Fahad I Askar, Mashael Y Altowairqi

**Affiliations:** 1 Oncology Center, Khalid University Hospital, Riyadh, SAU; 2 Internal Medicine, Khalid University Hospital, Riyadh, SAU

**Keywords:** prevalance, internal medicine, b-symptoms, survival rate, lymphoma, bone marrow, hodgkin

## Abstract

Objectives

To estimate the current prevalence of bone marrow involvement in classical Hodgkin lymphoma (HL) patients diagnosed at King Khalid University Hospital (KKUH), Riyadh, Saudi Arabia.

Methods

A cross-sectional study was conducted among classical Hodgkin’s lymphoma patients, diagnosed between 2015 and 2021 at KKUH. We retrospectively collected clinical and pathological information from all adult patients aged 18 years or older with a diagnosis of HL. Survival analyses were performed using the log-rank test and Kaplan-Meier curves.

Results

The study included 140 patients, 60 (42.86%) of whom were female. Bone marrow involvement was seen in 15 (10.71%) patients, 58 (41.43%) patients had an advanced-stage disease, and 20 (14.29%) patients had gastrointestinal involvement. Patients with bone marrow involvement had a median survival of 71 months (95% confidence interval (CI): 16.7-125.3) compared to patients without bone marrow involvement who had a median survival of 68 months (95% CI: 50.7-85.3).

Conclusion

The prevalence of bone marrow involvement in HL patients, as well as the proportion of patients presenting with advanced disease at the time of diagnosis, was higher compared to Western data. This could be attributed to a delay in diagnosis or more aggressive disease biology.

## Introduction

Hodgkin lymphoma (HL) is a lymphoid tumor derived from B-lymphocytes, histologically characterized by the presence of Hodgkin and Reed-Sternberg cells. It usually spreads to adjacent nodal sites but rarely affects other tissues. Examples of extra-nodal sites include the lungs, liver, and bone or bone marrow (BM) [1].

The BM is a major site of lymphomatous cell aggregation; thus, BM evaluation is an important part of HL staging. Ann-Arbor staging, which is commonly used in lymphoma, considers BM involvement a high disease stage (stage IV) [2-4]. According to Vassilakopoulos et al., upstaging of HL after BM studies showed therapeutic and prognostic significance [5].

There is limited information about patients with HL and its relationship with BM involvement in Saudi Arabia. Therefore, our study aimed to estimate the prevalence of BM involvement in HL patients in Saudi Arabia and to compare the results with published data from the Western world. The secondary objective was to estimate the overall survival in these patients.

## Materials and methods

This quantitative retrospective study was conducted among classical HL patients between 2015 and 2021 at King Khalid University Hospital (KKUH), Riyadh, Saudi Arabia. A total of 140 patients (n=140) who presented to our center with classical HL were included in this study. Other lymphoma subtypes were excluded, and survival outcome measures were determined using the log-rank test and summarized as Kaplan-Meier curves. Descriptive statistics (frequencies, percentages, medians, and ranges) were used to describe categorical and quantitative variables. Data were collected from hospital records, ensuring anonymity and the privacy and confidentiality of participants’ information. Patient consent was also obtained. Data were analyzed using SPSS 21.0. (IBM Corp, Armonk, NY). The study was approved by the local ethics board (number E-18-3624, dated 17/10/2021).

## Results

Of the 140 patients included in this study, 80 (57.14%) were men and 60 (42.86%) were women. Age ranged between 14 and 71 years, with a median age of 30 years. B symptoms were observed in 54 patients (38.57%). Overall, 30 (21.34%) patients had limited stage (1-2), 58 (41.43%) patients had advanced stage (3-4), and 52 (37.14%) patients were insufficient to stage (Table 1).

**Table 1:** Baselines characteristics of included patients (n=140) NA= Not available, LDH= Lactate dehydrogenase, WBC= White blood cells, Hb= Hemoglobin

Variable		Number (%)
Gender	Male	80 (57.14%)
	Female	60 (42.86%)
	Total	140 (100%)
B symptoms	Present	54 (38.57%)
	Absent	34 (24.29%)
	Insufficient	52 (37.14%)
	Total	140 (100%)
Ann-Arbor stage	Limited	30 (21.34%)
	Advanced	58 (41.43%)
	Insufficient	52 (37.14%)
	Total	140 (100%)
Bone Marrow involvement	Yes	15 (10.71%)
	No	97 (69.29%)
	Not Done	28 (20%)
	Total	140 (100%)
Gastrointestinal involvement	Yes	20 (14.29%)
	No	120 (85.71%)
	Total	140 (100%)
LDH	Above normal	24 (17.14%)
	Normal	29 (20.71%)
	NA	87 (62.14%)
Biopsy site	Extranodal	10 (7.14%)
	Nodal	129 (92.14%)
	Not documented	1 (0.71%)
	Total	140 (100%)
Histological diagnosis	Classical	120 (85.71%)
	Non-Classical	8 (5.71%)
	Not documented	12 (8.57%)
	Total	140 (100%)
Status of all HL patients	Alive	123 (87.85%)
	Passed away	17 (12.14%)
	Total	140 (100%)
Variable		Median (range)
Age at diagnosis		30 (14-71) years
LDH		262.51 (89-862) unit/L
Platelet		341 (40-899) x10^9/L
WBC		9.58 (2.7-24.7) x10^9/L
Hb		113 (12-168) g/L

Out of 112 BM biopsies, 15 (10.71%) showed lymphoma infiltration and 97 (69.29%) were negative.Twenty-eight (20%) patients did not undergo BM biopsy. Gastrointestinal involvement was positive in 20 (14.29%) and negative in 120 (85.71%) patients. Lactate dehydrogenase (LDH) levels were above normal in 24 (17.14%) patients, normal in 29 (20.71%) patients, and there was no information for 87 (62.14%) patients. For 76 patients evaluated using available data, LDH ranged between 89 and 862 u/I (median: 262.51 u/I), platelet count ranged between 40 and 899 x 10^9/L (median: 341 x 10^9/L), white blood cells ranged between 2.7 and 24.7 x 10^9/L (median: 9.58 x 10^9/L), hemoglobin ranged between 12 and 16.8 g/dl (median: 11.3 g/dl), and lymphocyte count ranged between 0.2 and 4.5 x 10^9/L (median: 1.6 x 10^9/L) (Table 1).

The biopsies were taken from extranodal sites in 10 (7.14%) patients and nodal sites in 129 (92.14%) patients. One biopsy (0.71%) was not documented. The biopsies were histologically diagnosed as classical HL in 120 (85.71%) patients and non-classical HL in eight (5.71%) patients. Twelve (8.57%) patients were not documented (Table 1). The status of all HL patients was 123 (87.85%) alive and 17 (12.14%) passed away (Table 1).

**Figure 1:**
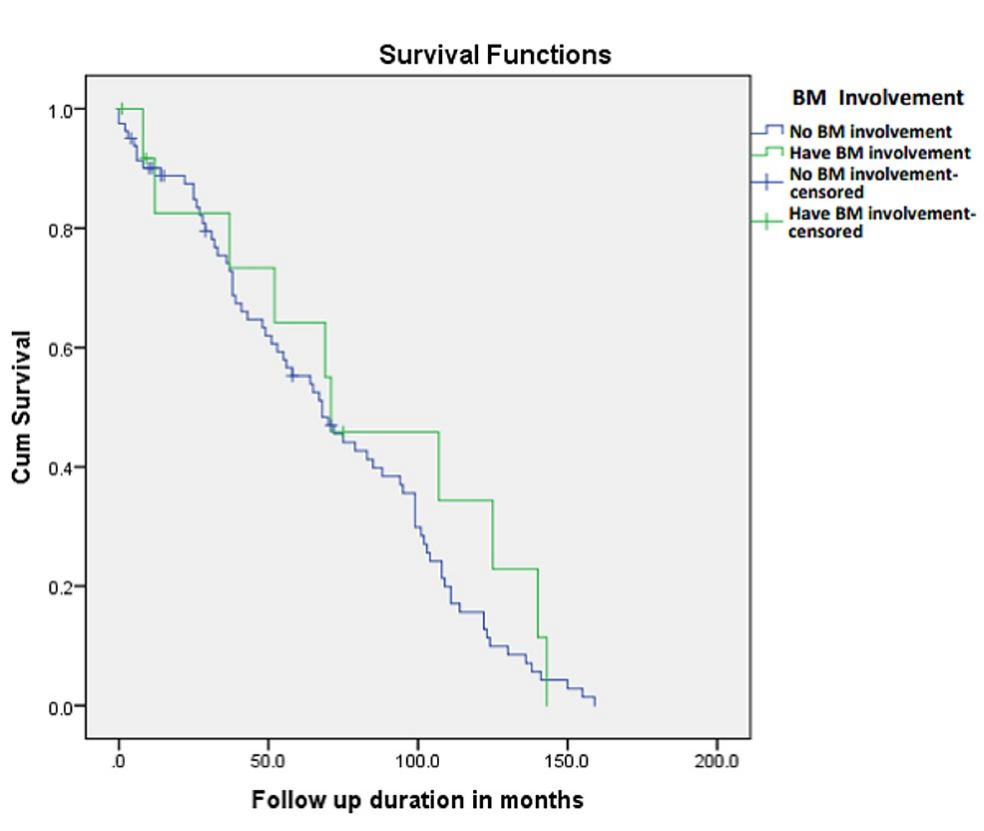
Kaplan-Meier survival curve Kaplan-Meier curve for survival analysis between patients with or without bone marrow involvement

While Table 1 summarizes the baseline characteristics of the included patients, Table 2 summarizes the mean and median survival rate in all HL patients with or without BM involvement and overall. Figure 1 shows the Kaplan-Meier curve for overall survival.

**Table 2:** Median and mean follow-up in months (survival rate) of all Hodgkin’s lymphoma patients

Variables	Mean				Median			
	Estimate	Std. Error	95% Confidence Interval		Estimate	Std. Error	95% Confidence Interval	
			Lower Bound	Upper Bound			Lower Bound	Upper Bound
No bone marrow involevment	70.741	4.975	60.989	80.492	68.000	8.837	50.679	85.321
Have bone marrow involvement	81.769	15.218	51.941	111.597	71.000	27.697	16.714	125.286
Overall	72.055	4.720	62.804	81.305	69.000	5.471	58.277	79.723

There was no statistically significant difference in survival between patients with or without BM involvement as can be seen in Table 2. Patients with BM involvement had a median survival of 71 months (95% confidence interval (CI) of 16.7-125.3) compared to patients without BM involvement who had a median survival of 68 months (95% CI: 50.7-85.3) (Table 2).

## Discussion

Our study aimed to estimate the BM involvement in patients diagnosed with HL in our region, due to the limited local information available on this topic. We showed that BM involvement was estimated to be around 10.71%, which is higher than that reported in Western populations. Western literature estimates that around 5-6% of patients with HL have BM involvement [5-6]. It is also important to note that most of our patients presented with advanced disease, contrary to the Western population [6]. Patients may present late due to a delay in seeking medical treatment in our region or it could simply reflect different disease biology. In another study conducted in Pakistan, Lone et al. [7] showed that the proportion of BM involvement was higher (38%) than our results [5-10]. It is hypothesized that the rates in developing countries are higher than those in developed countries. Similarly, another study conducted in Egypt showed that out of all the classical HL cases, 28.57% had BM infiltration [11]. Moreover, it was noted in our study that the prevalence of B symptoms in our region was 38.57%; in contrast, the prevalence of B symptoms in other regions was lower [6-7].

Most of our patients were diagnosed under 30 years of age with a median age of 30 years, which is similar to studies around the world reporting a median age of around 30 years [5-10].

Interestingly, 80 (57.14%) patients were males, and it is predominantly noted that HL is frequently reported in male patients. A higher male-to-female ratio has also been reported in other studies [5,8].

We did not find a statistical difference in survival between patients with or without BM involvement. However, our results should be interpreted with caution, as the study could be underpowered to show a statistical difference because of the relatively small sample size.

The limitations of this study include its retrospective design and the relatively small sample size. However, since we included all patients with HL between the desired dates, the sample size could not have been increased further.

## Conclusions

The prevalence of BM involvement at the time of diagnosis of classical HL in our center was higher than expected, as was the proportion of patients presenting with advanced disease. This could be due to a delay in diagnosis or more aggressive disease biology compared to data from studies performed in Western nations. Future studies with larger sample sizes are needed to confirm this finding and to determine the reasons for higher-risk patients in our region.
